# Nomogram for prediction of recurrence in patients with lumbar disc herniation after unilateral biportal endoscopy spinal surgery: a retrospective study

**DOI:** 10.3389/fsurg.2025.1564825

**Published:** 2025-06-16

**Authors:** Yi Rong, Kaixuan Wang, Yalan Pan, Tianchi Zhang, Yong Ma, Lining Wang, Yang Guo, Si Chen, Yang Shao, Tingchen Zhu, Shixiang Wu, Zhen Hua, Jianwei Wang, Hao Yu

**Affiliations:** ^1^ Department of Traumatology & Orthopedics, Wuxi Affiliated Hospital of Nanjing University of Chinese Medicine, Wuxi, China; ^2^ School of Integrative Medicine, Nanjing University of Chinese Medicine, Nanjing, Jiangsu, China; ^3^ Laboratory of New Techniques of Restoration & Reconstruction, Institute of Traumatology & Orthopedics, Nanjing University of Chinese Medicine, Nanjing, China; ^4^ School of Nursing, Nanjing University of Chinese Medicine, Nanjing, Jiangsu, China; ^5^ Yancheng Hospital of Traditional Chinese Medicine, Yancheng Affiliated Hospital of Nanjing University of Chinese Medicine, Yancheng, China; ^6^ Jiangsu CM Clinical Innovation Center of Degenerative Bone & Joint Disease, Wuxi Affiliated Hospital of Nanjing University of Chinese Medicine, Wuxi, Jiangsu, China; ^7^ Department of Orthopedic Surgery, Nanjing Hospital of Chinese Medicine Affiliated to Nanjing University of Chinese Medicine, Nanjing, China

**Keywords:** LDH, UBE, recurrence, nomogram, prediction model

## Abstract

**Objective:**

This study aimed to construct a nomogram to predict the likelihood of early recurrence in patients with lumbar disc herniation (LDH) following unilateral biportal endoscopic (UBE) surgery.

**Methods:**

A retrospective analysis was conducted on LDH patients who underwent UBE surgery in our department between January 1, 2022, and December 31, 2023. The eligible cohort was randomly divided into training and validation sets in a 7:3 ratio. Key predictors for the nomogram were identified through a combination of least absolute shrinkage and selection operator (LASSO) regression and multivariate logistic regression analysis. The model's performance was assessed using the C-index, the area under the receiver operating characteristic curve (AUC), calibration curves, and decision curve analysis. The validation set was used to further evaluate the model's robustness.

**Results:**

A total of 289 patients were included in the study, among whom 50 experienced recurrent LDH (rLDH). Five risk factors were identified as significant predictors for rLDH: width of protrusion base (WPB), bone removal range (BRR), Modic changes, type of LDH, and middle vertebral space height (MVH). The C-index values for the training and validation sets were 0.834 and 0.804, respectively. The AUC values were 0.834 (95% CI: 0.750–0.918) in the training set and 0.804 (95% CI: 0.697–0.910) in the validation set. Calibration curves demonstrated excellent concordance between the predicted and observed outcomes. Decision curve analysis indicated that using the nomogram to predict rLDH risk provided a positive net benefit when the threshold probability was between 4% and 63%.

**Conclusion:**

This study successfully developed and validated a nomogram to predict early recurrence in LDH patients following UBE surgery. The model provides a valuable tool for clinicians to assess individual rLDH risk, enabling timely interventions to improve postoperative outcomes.

## 1 Introduction

Lumbar disc herniation (LDH) is a prevalent clinical condition that often manifests as pain and numbness in the lower back and legs, significantly impairing patients' daily activities (1). Unilateral biportal endoscopic (UBE) surgery has become a widely adopted clinical approach due to its numerous advantages, including minimal trauma, a clear surgical field, reduced bleeding, low risk of nerve injury, low infection rates, and rapid recovery (2). However, some patients remain at risk of recurrent lumbar disc herniation (rLDH).

Recurrent LDH is defined as herniation at the same vertebral level causing symptomatic compression, irrespective of the time interval since the initial surgery (3). The recurrence of LDH may be attributed to factors such as nerve element compression by scar tissue during discectomy, residual disc fragments, or reactive tissues such as portions of the disc endplate, vertebrae, or fibrous rings (4). Reported recurrence rates range from 5%–18% among patients with LDH following their initial surgery (5). Recurrence exacerbates lower back and limb pain, with severe cases necessitating a second surgical intervention (6).

A second surgery often poses challenges due to fibrosis and scarring at the operative site caused by the primary procedure, making minimally invasive revision surgery more difficult. Additionally, recurrence imposes significant physical, psychological, and financial burdens on patients while straining medical resources. Hence, the prevention and early identification of rLDH are critical.

This study aims to establish and validate a predictive model to assess the early risk of developing rLDH after UBE surgery in LDH patients. By offering a user-friendly nomogram, we aim to enable clinicians to easily estimate individual recurrence risk, facilitating timely interventions and improving patient outcomes.

## 2 Design and methods

### 2.1 Research population

To ensure statistical robustness, *a priori* sample size calculation was performed using the formula for logistic regression:

$$N=\frac{\left(Z_{\alpha /2}+Z_{\beta })\times p(1-p\right)}{\delta^{2}}$$

where Z*_α_*_/2_ = 1.96 (95% confidence level), Z*_β_* = 0.84(80% power), *P* *=* *0.15* expected recurrence rate from prior literature (5, 6), and *δ* = 0.05 (margin of error). This yielded a minimum sample size of 246. To account for potential data loss and enhance model generalizability, 289 patients were ultimately enrolled.

This retrospective study included data from 289 patients diagnosed with lumbar disc herniation (LDH) who underwent unilateral biportal endoscopic (UBE) surgery in the Spinal Department of Wuxi Affiliated Hospital of Nanjing University of Traditional Chinese Medicine between January 1, 2022, and December 31, 2023. Among these, 50 patients experienced recurrence of LDH (rLDH), while the remaining 239 did not. Recurrence was assessed through clinical and imaging follow-up at 12 months postoperatively. Patients with unresolved symptoms underwent additional MRI scans to confirm recurrence. The study was approved by the Ethics Review Committee of the Wuxi Affiliated Hospital of Nanjing University of Chinese Medicine.

### 2.2 Inclusion and exclusion criteria

**Inclusion criteria:**

1. Age between 17 and 87 years.
2. Underwent unilateral two-channel spinal endoscopy.
3. First-time surgery.
4. Availability of complete clinical data.

**Exclusion criteria:**

1. History of lumbar surgery or contraindications to surgery or anesthesia.
2. Presence of lumbar tumors.
3. Diagnosis of mental illness.
4. Severe heart, liver, or kidney dysfunction.
5. Lumbar spine fractures, malformations, or tuberculosis.

**Figure 1:**
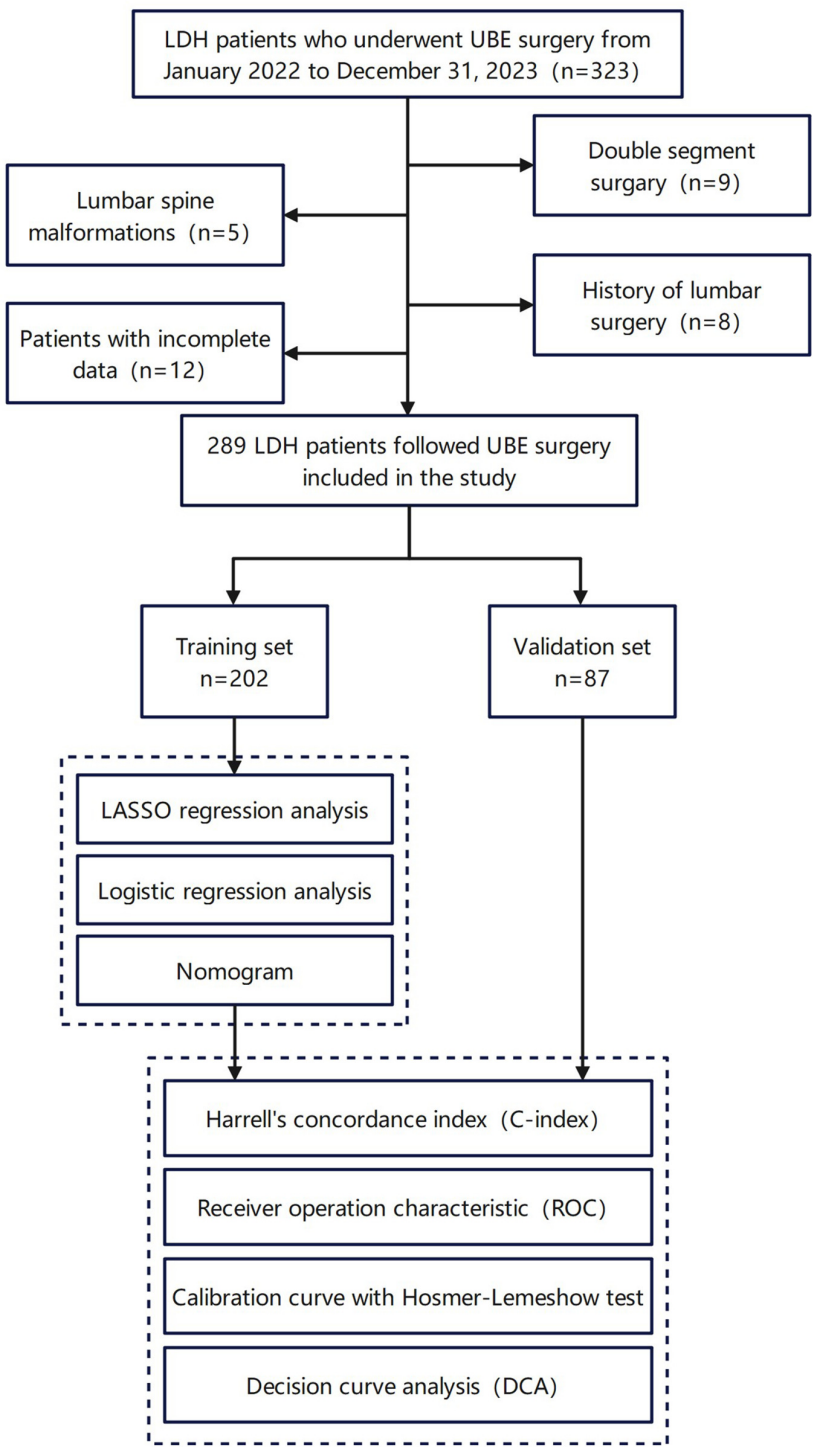
The design process of the research. This figure includes the inclusion and exclusion of patients, as well as the final number and grouping of study participants.

The study design and patient selection process are illustrated in Figure 1.

### 2.3 Data collection and potential predictors

The dataset comprised the following three categories of clinical parameters:

#### 2.3.1 Patient baseline characteristics

Sex, age, height, weight, body mass index (BMI), osteoporosis, hypertension, diabetes, hyperlipidemia, disease duration, postoperative time to ambulation (PLOB), and length of hospital stay (DIH).

#### 2.3.2 Operative data

Surgical segment, bone removal range (BRR), and operation time (OT). BRR was quantified as the percentage of the superior articular process resected during surgery (low resection: <50%) using pre- and postoperative CT scans.

#### 2.3.3 Imaging parameters

LDH Type: Classified as protrusion (contained) or prolapse (non-contained) based on the Michigan State University (MSU) classification criteria.Width of Protrusion Base (WPB): Maximum transverse diameter of the herniated disc base on axial MRI.Middle Vertebral. Height (MVH): Distance between midpoints of adjacent vertebral endplates on sagittal MRI.

Disc diameter (DD), vertebral canal diameter (VCD), and diameter ratio (DD/VCD). Degeneration and Stability Assessments: Lumbar instability, Pfirrmann degeneration grade (PC), fat infiltration classification (FIC), and lumbar lordotic angle (LLA). Modic changes were classified as Type III based on MRI criteria (vertebral endplate sclerosis without bone marrow edema).

### 2.4 Logistic regression analysis and nomogram development

The 289 patients were randomly divided into training and validation sets in a 7:3 ratio (7, 8). No significant differences were observed in demographic or clinical characteristics between the two groups. LASSO regression analysis was performed on the training set to identify potential predictors, effectively eliminating variables with minimal correlation or multicollinearity to address high-dimensional data issues (9). Multivariate logistic regression analysis was then applied to identify five key predictive variables, which were used to construct a nomogram based on the training set. The model was subsequently validated using the validation set.

### 2.5 Model performance and validation

Model performance was assessed using discrimination and calibration metrics (10). Discrimination was quantified using Harrell's concordance index (C-index) and receiver operating characteristic (ROC) curve analysis, with an AUC value above 0.80 considered indicative of good discrimination (11–13). Calibration was evaluated using calibration curves and the Hosmer-Lemeshow test, assessing the agreement between predicted and observed rLDH occurrence rates (14). Decision curve analysis (DCA) was conducted to evaluate the model's clinical utility and net benefit (15). All assessments were performed using bootstrap validation with 1,000 resamples.

### 2.6 Statistical analysis

Statistical analyses and data visualization were performed using R software version 4.4.1 (The R Project for Statistical Computing, https://www.r-project.org). Continuous variables with a normal distribution were expressed as mean ± standard deviation (SD), while those with a skewed distribution were presented as the median [M] and interquartile range [Q25–Q75]. Independent *t*-tests were used for group comparisons of continuous data with equal variance, while unequal variance *t*-tests were applied for datasets with unequal variances. Model fit was evaluated using the Hosmer-Lemeshow goodness-of-fit test. Model performance was assessed using discrimination metrics (C-statistic) and calibration curves. To mitigate overfitting and quantify optimism, the nomogram underwent internal validation with bootstrap resampling (1,000 iterations), and an optimism-corrected C-statistic was calculated. Clinical validity and net benefit of the nomogram were further assessed using DCA (15).

Logistic regression analysis and nomogram development were performed using R packages including rms, pROC, forestplot, corrplot, glmnet, caret, CBCgrps, nortest, ggpubr, compareGroups, regplot, ggplot2, and rmda. Bilateral *p*-values < 0.05 were considered statistically significant.

## 3 Results

### 3.1 Patient characteristics

As shown in Table 1, this study included 289 eligible patients, who were randomly assigned to the training set (*n* = 202) and validation set (*n* = 87). Among them, 50 patients developed recurrent lumbar disc herniation (rLDH) postoperatively, with 32 in the training set and 18 in the validation set. Statistical analysis revealed no significant differences between the training and validation sets in baseline characteristics (*P* > 0.05). Details of the baseline characteristics are presented in Table 2.

**Table 1:** Baseline characteristics between the recurrent and nonrecurrent groups.

Variables	Total	Non recurrent	Recurrent	*p*
	(*n* = 289)	(*n* = 239)	(*n* = 50)	
Osteoporosis, *n* (%)				0.551
No	231 (80)	189 (79)	42 (84)	
Yes	58 (20)	50 (21)	8 (16)	
Hypertension, *n* (%)				0.076
No	204 (71)	163 (68)	41 (82)	
Yes	85 (29)	76 (32)	9 (18)	
Diabetes, *n* (%)			0.554	
No	256 (89)	210 (88)	46 (92)	
Yes	33 (11)	29 (12)	4 (8)	
Hyperlipidemia, *n* (%)				0.140
No	236 (82)	191 (80)	45 (90)	
Yes	53 (18)	48 (20)	5 (10)	
Segments, *n* (%)				0.506
L1/2	1 (0)	1 (0)	0 (0)	
L2/3	2 (1)	2 (1)	0 (0)	
L3/4	14 (5)	10 (4)	4 (8)	
L4/5	134 (46)	108 (45)	26 (52)	
L5/S1	138 (48)	118 (49)	20 (40)	
Protrusion Site, *n* (%)				0.158
1	73 (25)	55 (23)	18 (36)	
2	170 (59)	147 (62)	23 (46)	
3	45 (16)	36 (15)	9 (18)	
4	1 (0)	1 (0)	0 (0)	
Pfirrmann, *n* (%)				0.139
1	1 (0)	1 (0)	0 (0)	
2	24 (8)	21 (9)	3 (6)	
3	83 (29)	65 (27)	18 (36)	
4	133 (46)	107 (45)	26 (52)	
5	48 (17)	45 (19)	3 (6)	
Fatty infiltration, *n* (%)				0.055
1	110 (38)	92 (38)	18 (36)	
2	154 (53)	122 (51)	32 (64)	
3	22 (8)	22 (9)	0 (0)	
4	3 (1)	3 (1)	0 (0)	
Modic Change, *n* (%)				0.003
No	213 (74)	185 (77)	28 (56)	
Yes	76 (26)	54 (23)	22 (44)	
Lumbar Instability, *n* (%)				0.903
No	244 (84)	201 (84)	43 (86)	
Yes	45 (16)	38 (16)	7 (14)	
BRR, *n* (%)				0.006
No	141 (49)	126 (53)	15 (30)	
Yes	148 (51)	113 (47)	35 (70)	
LDH type, *n* (%)				0.054
Protrusion	176 (61)	139 (58)	37 (74)	
Prolapse	113 (39)	100 (42)	13 (26)	
Sex, *n* (%)				0.522
Male	182 (63)	153 (64)	29 (58)	
Female	107 (37)	86 (36)	21 (42)	
Age	51 (40, 63)	52 (40.5, 63.5)	49 (40, 60.5)	0.489
Weight, kg	69 (60, 77)	70 (61, 77.5)	65 (60, 76.75)	0.279
Height, cm	170 (160, 174)	170 (160, 175)	170 (161, 172)	0.780
BMI, kg/m^2^	24.22 (22.39, 26.56)	24.34 (22.49, 26.5)	23.55 (21.73, 26.55)	0.223
Disease Course, month	3 (1, 6)	3 (1, 7)	2 (1, 4)	0.204
Lordosis Angle	7 (4, 11)	8 (4, 11)	6 (2.25, 11)	0.238
MVH, Mean ± SD	9.58 ± 2.12	9.39 ± 2.05	10.49 ± 2.19	0.002
WPB, mm	14.58 (12.15, 16.82)	14.21 (11.50, 15.93)	17.16 (15.23, 18.98)	<0.001
DD, Mean ± SD	36.53 ± 3.67	36.45 ± 3.56	36.91 ± 4.17	0.477
VCD, Mean ± SD	17.71 ± 2.46	17.61 ± 2.46	18.18 ± 2.45	0.145
Diameter Ratio, Mean ± SD	0.49 ± 0.09	0.49 ± 0.09	0.5 ± 0.09	0.626
OT, min	85 (66, 105)	82 (65 105)	85 (75, 115)	0.183
PLOB, day	3 (3, 5)	3 (3, 5)	4 (3, 6)	0.079
DIH, day	6 (5, 8)	6 (5, 8)	7 (6, 9)	0.119

**Table 2:** Baseline characteristics of participants in training and validation set.

Variables	Training set (*n* = 202), *N* (%)	Validation set (*n* = 87), *N* (%)	*P*
Osteoporosis			0.343
No	158 (78.2)	73 (83.9)	
Yes	44 (21.8)	14 (16.1)	
Hypertension			0.980
No	142 (70.3)	62 (71.3)	
Yes	60 (29.7)	25 (28.7)	
Diabetes			0.563
No	177 (87.6)	79 (90.8)	
Yes	25 (12.4)	8 (9.20)	
Hyperlipidemia			0.630
No	163 (80.7)	73 (83.9)	
Yes	39 (19.3)	14 (16.1)	
Segment			0.527
L1/2	1 (0.50)	0 (0.00)	
L2/3	1 (0.50)	1 (1.15)	
L3/4	12 (5.94)	2 (2.30)	
L4/5	90 (44.6)	44 (50.6)	
L5/S1	98 (48.5)	40 (46.0)	
Protrusion Site			0.635
1	40 (19.8)	33 (37.9)	
2	129 (63.9)	41 (47.1)	
3	33 (16.3)	12 (13.8)	
4	0 (0.00)	1 (1.15)	
Pfirrmann			0.916
1	1 (0.50)	0 (0.00)	
2	18 (8.91)	6 (6.90)	
3	58 (28.7)	25 (28.7)	
4	90 (44.6)	43 (49.4)	
5	35 (17.3)	13 (14.9)	
Fatty infiltration			0.785
1	74 (36.6)	36 (41.4)	
2	109 (54.0)	45 (51.7)	
3	17 (8.42)	5 (5.75)	
4	2 (0.99)	1 (1.15)	
Modic Change			0.445
No	152 (75.2)	61 (70.1)	
Yes	50 (24.8)	26 (29.9)	
Lumbar Instability			0.080
No	176 (87.1)	68 (78.2)	
Yes	26 (12.9)	19 (21.8)	
BRR			0.433
No	95 (47.0)	46 (52.9)	
Yes	107 (53.0)	41 (47.1)	
LDHtype			0.689
Protrusion	120 (59.4)	56 (64.4)	
Prolapse	82 (40.6)	31 (35.6)	
Sex			0.650
Male	125 (61.9)	57 (65.5)	
Female	77 (38.1)	30 (34.5)	
Age	51.3 (14.8)	52.1 (13.5)	0.662
Weight	69.2 (13.3)	70.7 (12.2)	0.338
Height	168 (9.08)	169 (7.58)	0.540
BMI	24.4 (3.35)	24.8 (3.15)	0.385
Disease Course	11.4 (24.9)	11.4 (26.4)	0.997
Lordosis Angle	7.61 (5.11)	7.09 (4.73)	0.411
MVH	9.50 (2.17)	9.58 (2.03)	0.771
WPB	14.6 (3.21)	14.8 (3.79)	0.661
DD	36.6 (3.67)	36.5 (3.71)	0.847
VCD	17.5 (2.23)	18.1 (2.90)	0.096
Diameter Ratio	0.48 (0.08)	0.50 (0.10)	0.101
OT	86.7 (28.9)	90.1 (25.1)	0.306
PLOB	3.96 (1.92)	4.25 (2.19)	0.282
DIH	6.64 (1.86)	6.91 (1.94)	0.285

### 3.2 Identification of predictive factors

**Figure 2:**
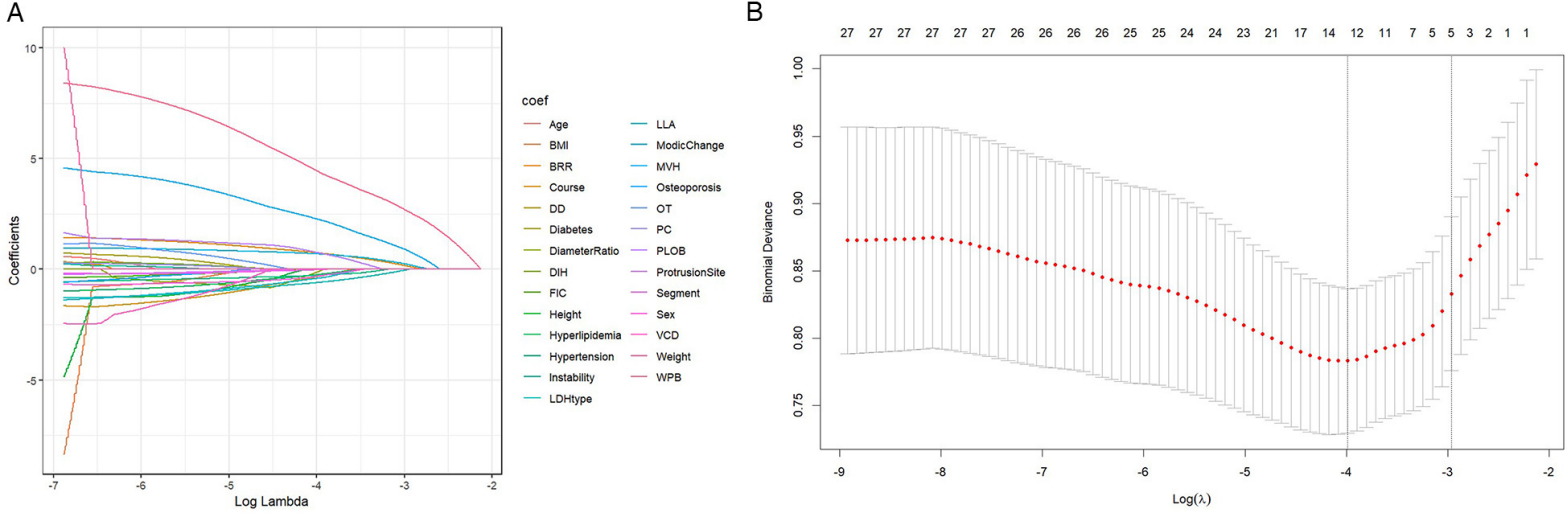
LASSO regression path diagram for variable selection. **(A)** Variable screening: The convergence point of the curve in the figure indicates the key variables selected, including WPB, BRR, etc. **(B)** Penalty coefficient: The *X*-axis shows the influence of the change of penalty coefficient on variable screening. The model prevents overfitting by controlling the penalty coefficient.

To identify the most relevant predictive factors, a two-step filtering process was employed. First, least absolute shrinkage and selection operator (LASSO) regression was performed, which helped to minimize overfitting and enhance model robustness by selecting six potential predictors: Middle Vertebral Height (MVH), Modic Change, Width of Protrusion Base (WPB), Bone Removal Range (BRR), and LDH Type (Figures 2A,B).

Subsequently, multivariate logistic regression analysis confirmed the independence of these predictors. Key findings included:

• MVH (OR = 0.30, 95% CI: 0.12–0.499, *P* = 0.001)
• Modic Change (OR = 1.16, 95% CI: 0.40–1.93, *P* = 0.003)
• WPB (OR = 0.30, 95% CI: 0.19–0.42, *P* < 0.001)
• BRR (OR = 1.22, 95% CI: 0.48–2.022, *P* = 0.002)
• LDH Type (OR = −1.04, 95% CI: −1.86 to −0.28, *P* = 0.009).

**Figure 3:**
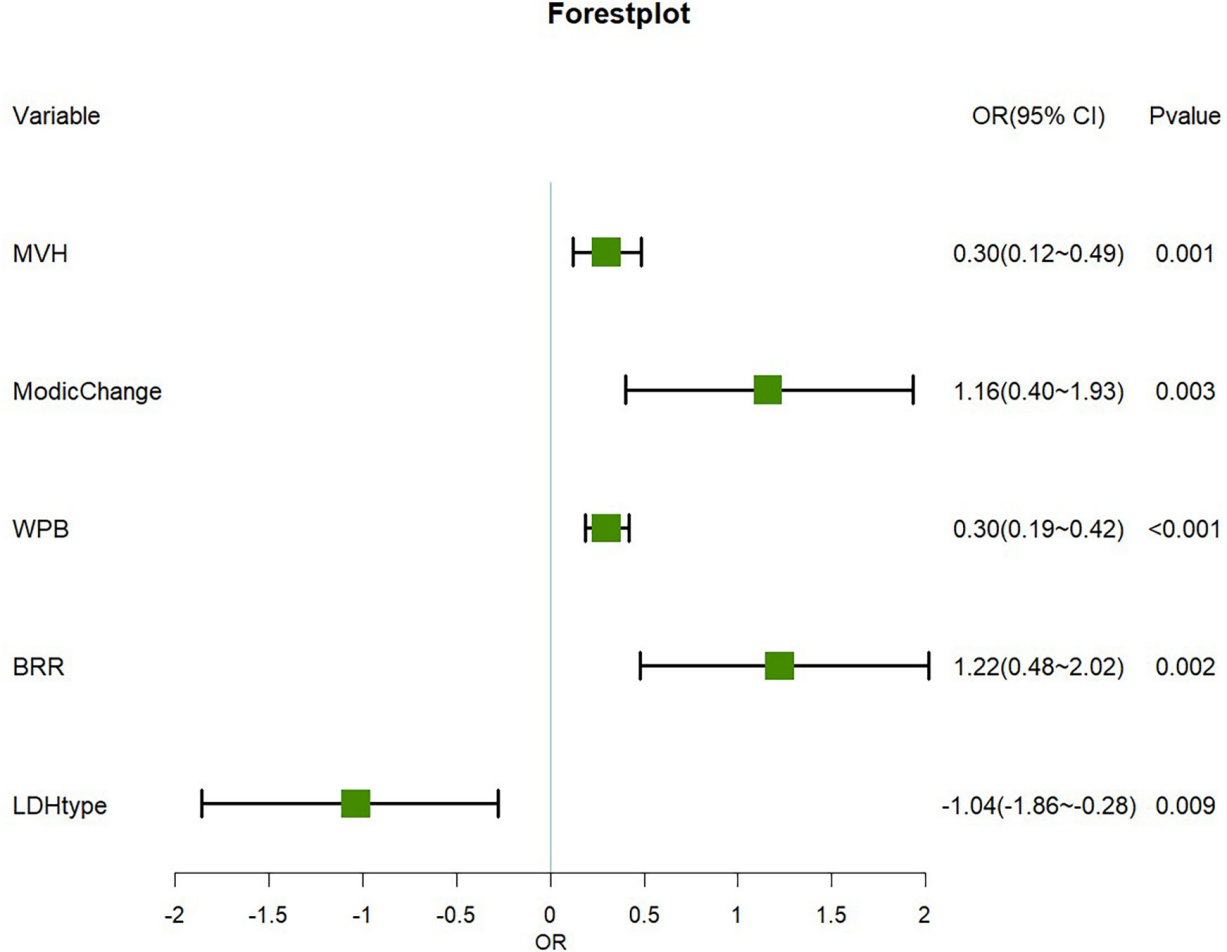
Forest plot of the main predictor variables. Important risk factors: WPB, BRR, Modic changes and MVH significantly increase the risk of recurrence (OR > 0). Protective factor: The LDH type was determined as the protective factor (OR < 0).

A nomogram was subsequently developed to predict the probability of rLDH using these five predictive variables (Figure 3).

### 3.3 Development of an individualized prediction model

**Figure 4:**
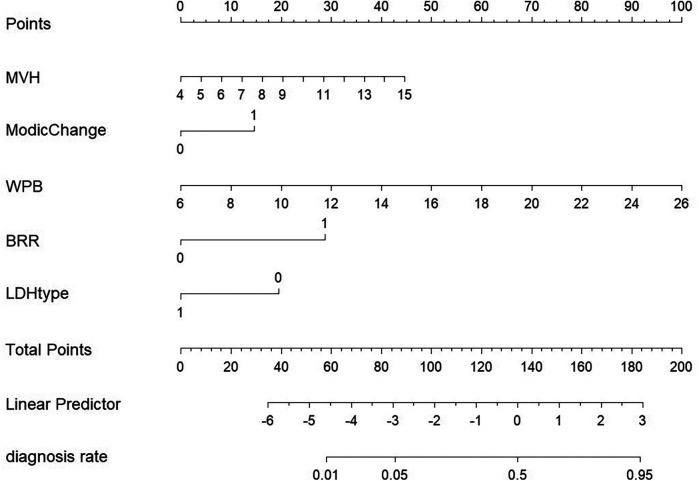
Column diagram model. A cumulative score helps doctors assess individual risk. Risk factors include widthprotruded base (WPB), extent of bone resection (BRR), Modic changes, type of lumbar disc herniation (LDH type), and middle vertebral height (MVH).

The nomogram integrated the identified predictors and revealed their relative contributions to the risk of rLDH. Among the predictors, WPB emerged as the strongest risk factor, followed by MVH, Modic Change, and BRR. On the other hand, disc prolapse were identified as protective factors. The nomogram provides a personalized assessment of recurrence risk, facilitating clinical decision-making (Figure 4).

### 3.4 Predictive model validation

#### 3.4.1 Discrimination

**Figure 5:**
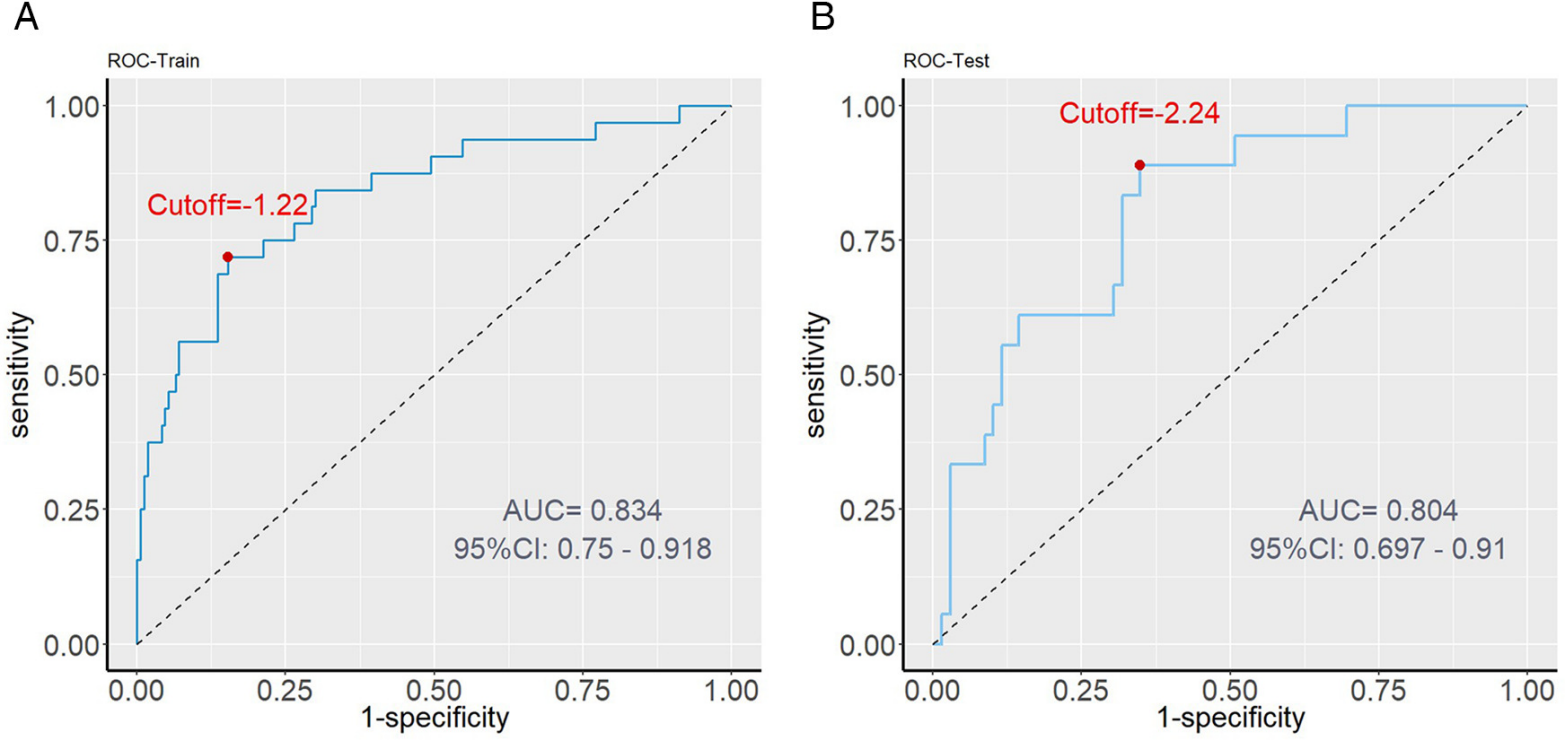
ROC curve analysis. **(A)** Training set performance: The AUC value is 0.834, indicating that the model has high predictive ability in internal data. **(B)** Validation set performance: AUC value of 0.804 proves the robustness of the model in external data.

The model demonstrated strong discriminative ability, as indicated by C-index values of 0.834 for the training set and 0.804 for the validation set. Additionally, area under the curve (AUC) values were 0.834 (95% CI: 0.750–0.918) in the training set and 0.804 (95% CI: 0.697–0.910) in the validation set, further supporting the model's robust discrimination (Figures 5A,B).

#### 3.4.2 Calibration

**Figure 6:**
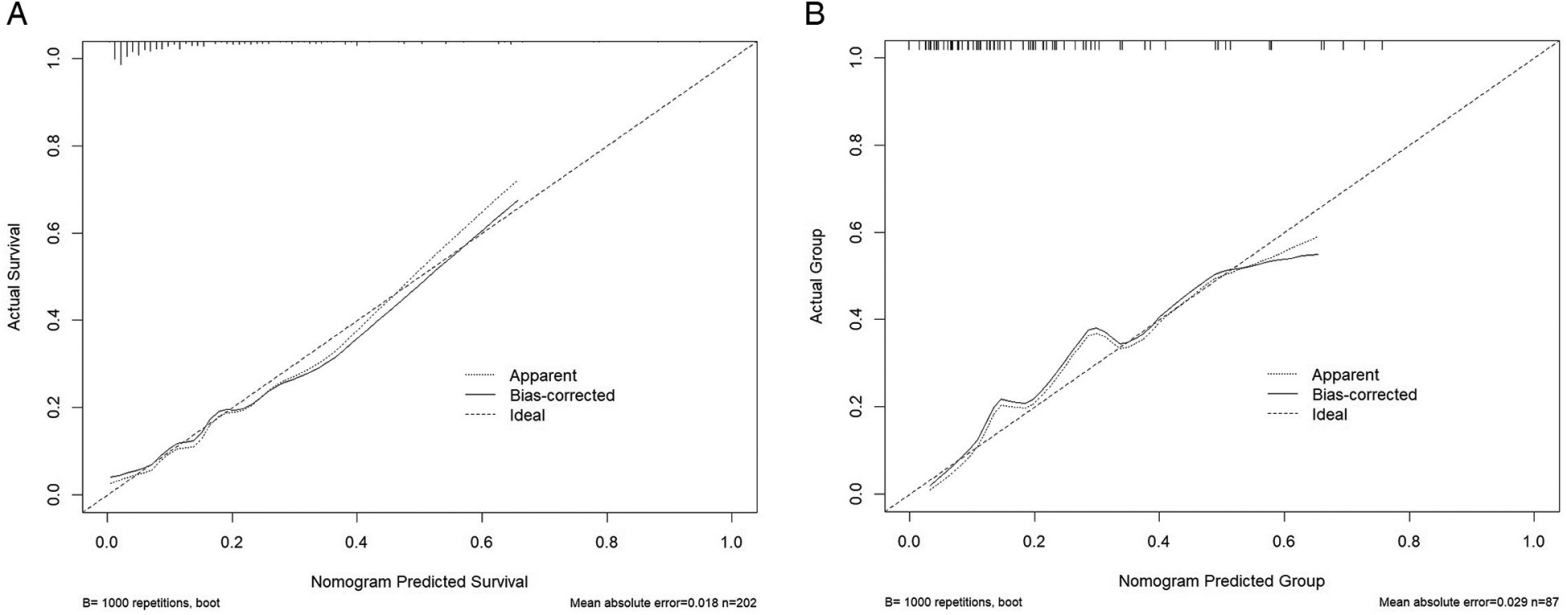
Calibration curve. **(A)** Training set calibration: The training set curve is close to the diagonal, indicating that the predicted value is highly consistent with the actual value. **(B)** Verification set calibration: The verification set curve further proves that the model has good calibration ability.

The calibration curves showed excellent agreement between predicted and actual probabilities of rLDH occurrence in both the training (*χ*² = 7.92, df = 8, *P* = 0.442) and validation sets (*χ*² = 7.90, df = 8, *P* = 0.444). These results indicate that the model is well-calibrated (Figure 6).

#### 3.4.3 Clinical application

**Figure 7:**
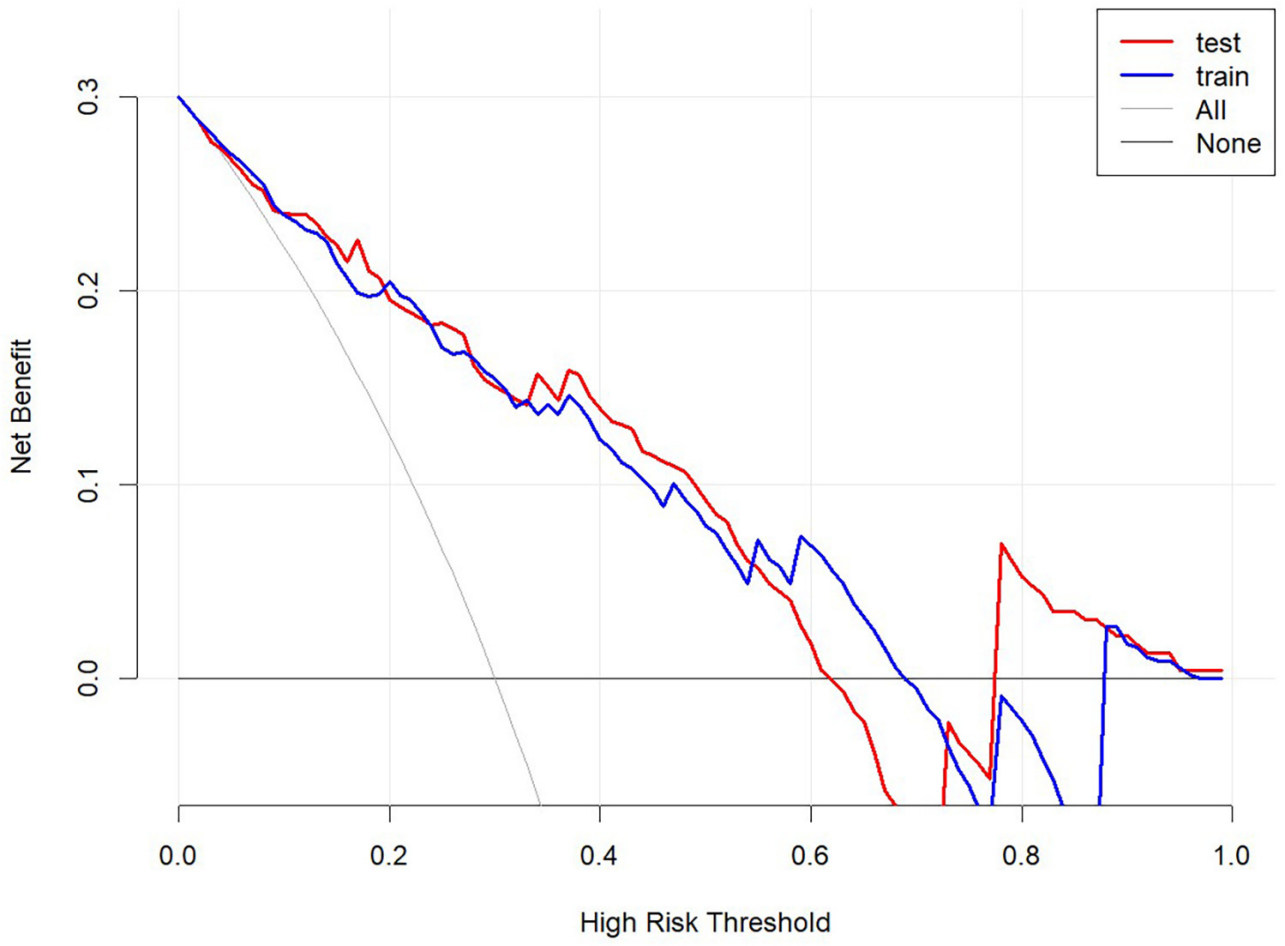
Decision curve analysis. Net benefit curve: Within the threshold probability range of 4%–63%, the model provides A higher net benefit than no intervention or full intervention strategies. Baseline comparison: The baseline comparison indicates that the model forecasts better and can effectively reduce unnecessary interventions.

Decision curve analysis (DCA) demonstrated that the nomogram provides significant net benefits for predicting rLDH across a wide range of risk thresholds (4%–63%) in both the training and validation sets. This suggests the nomogram's practical utility in guiding clinical interventions (Figure 7).

## 4 Discussion

The causes of recurrent lumbar disc herniation (rLDH) following endoscopic surgery remain a topic of considerable debate (16, 17). While previous studies have examined a wide range of factors associated with rLDH, the conclusions often vary. For instance, Guray Bulut et al. found that variables such as gender, age, diabetes mellitus (DM), obesity, surgical level, disc degeneration, and disc type were not significantly associated with rLDH, though hypertension (HT) appeared more prevalent in recurrent cases (18). Mengxian Jia, using a directed mutation-guided SVM model, highlighted factors like herniated disc level, Modic changes, disc height, disc length, and disc width as critical predictors for rLDH (19). Furthermore, multivariate logistic regression analysis by other researchers showed that comorbid diabetes and smoking significantly increased the risk of recurrence (20). Despite these findings, many of these factors remain insufficiently validated, necessitating further investigation.

To address this gap, we developed and validated the first nomogram specifically designed to assess rLDH risk in lumbar disc herniation (LDH) patients undergoing unilateral biportal endoscopic (UBE) surgery. By incorporating key clinical features—WPB, BRR, Modic Change, LDH Type, and MVH—this model demonstrated excellent predictive performance and offers clinicians a valuable tool for early intervention and risk mitigation.

In this study, WPB emerged as the strongest risk factor. A larger protrusion base indicates more extensive annulus fibrosus damage, increasing the likelihood of residual nucleus pulposus fragments after surgery. This often necessitates more aggressive annulus removal to achieve decompression, which may, in turn, exacerbate annular tears and defects. These defects not only accelerate disc degeneration but also contribute to nerve root adhesion and aseptic inflammation, both of which are significant contributors to chronic postoperative low back pain (21).

Similarly, LDH Type was identified as a critical predictor. Shan et al. reported that free disc herniation independently increases recurrence risk after lumbar discectomy (22), while Yurac et al. highlighted the role of non-encapsulated disc herniation and annular rupture in predicting rLDH (23). Yao et al. found that central disc herniation was associated with recurrence, likely due to challenges in adequately removing contralateral nucleus pulposus tissue (24). Although variations exist in reported findings, there is consensus that annular rupture and central or free disc herniation are significant risk factors for recurrence.

Our findings indicate that patients with higher MVH and more extensive BRR are at greater risk of early rLDH. The intervertebral discs and facet joints are critical for maintaining the biomechanical stability of the lumbar spine. Studies suggest that removing more than 50% of the superior facet joint during surgery significantly destabilizes the lumbar spine (25, 26). Furthermore, finite element analyses have shown that removing the base of the upper articular process increases facet joint stress more than removing its tip, particularly during lateral flexion, extension, and rotation (27). These findings underscore the importance of minimizing structural damage to preserve stability and reduce the risk of rLDH.

Type III Modic changes, characterized by sclerotic endplate alterations, may exacerbate disc degeneration through impaired nutrient supply, aligning with our findings. Modic Change is another key predictor of rLDH. It results from cartilage endplate fractures and inflammatory responses that impair the nutrient supply to the intervertebral disc, hindering annulus fibrosus repair and promoting disc degeneration (28, 29). Studies have confirmed Modic Change as a risk factor for postoperative recurrence, consistent with our findings (24, 30).

The developed model provides an effective tool for estimating the likelihood of early recurrence following single-segment UBE surgery in LDH patients. This predictive ability supports personalized patient management in several ways:

1. Preoperative Assessment: A thorough patient evaluation, including detailed history-taking and clinical examination, can identify high-risk patients. For such individuals, clinicians may consider more aggressive surgical approaches or alternative treatments to mitigate recurrence risks.
2. Risk Communication: By using the nomogram, clinicians can engage in informed discussions with patients and their families about surgical risks, outcomes, and postoperative expectations, improving shared decision-making and patient satisfaction.
3. Postoperative Monitoring and Intervention: High-risk patients can benefit from closer monitoring and early intervention strategies, potentially preventing recurrence and improving outcomes.

## 5 Limitations

However, the study had various limitations: Our model is trained and validated using data from a single center, and its generalization to other centers and regions needs to be clarified. Nevertheless, we have done our best to include variables that are easy to collect and to simplify the model to prevent overfitting. Of course, teams from other centers are also welcome to join our research and contribute more multi-center data to the research. The sample size of our study is still small. The maximum follow-up duration of 12 months may underestimate long-term recurrence rates. Future studies with extended follow-up periods are warranted to validate our findings. Although model performance is evaluated by training and validation sets, sample size may limit the generalization of results. As recognized, external validation provides a more rigorous assessment of model robustness. In contrast, noise or bias in the external validation data set may mask the actual model performance found through internal validation (31, 32). The study was conducted on patients from a single healthcare facility, and it remains challenging to eliminate the selection bias and information bias associated with a single-center sample. For example, excluding recurrent LDH cases likely underestimates the role of scar adhesion in long-term recurrence. Exclusive focus on Type III Modic changes limits generalizability to other subtypes. Further studies should explore the differential impacts of Modic I and II changes on recurrence risk (33, 34). Therefore, conducting multi-center and prospective cohort studies is essential for more thorough exploration. While using nomograms improves models' interpretability, machine learning models' interpretability remains challenging.

For non-technical people, such as clinicians and patients, the model's decision-making process may need to be more transparent. In the future, we aim to integrate machine learning models into electronic medical record systems. This integration will allow us to predict individual cases by extracting patient information and metrics. Subsequently, we plan to present the predictions directly to doctors and patients to improve the usability of the model.

## 6 Conclusion

This study identified five independent predictors of recurrent lumbar disc herniation (rLDH) in patients undergoing unilateral biportal endoscopic (UBE) surgery, culminating in the development of an innovative nomogram model. The model exhibited excellent internal and external validation performance, offering clinicians a reliable tool for identifying high-risk patients and enabling personalized care and targeted management strategies. However, further extensive research and multi-center validation are necessary to enhance the model's generalizability and robustness.

## Data Availability

The original contributions presented in the study are included in the article/Supplementary Material, further inquiries can be directed to the corresponding author.
